# Analysis of Dieback in a Coastal Pinewood in Campania, Southern Italy, through High-Resolution Remote Sensing

**DOI:** 10.3390/plants13020182

**Published:** 2024-01-09

**Authors:** Rosario Nicoletti, Luigi De Masi, Antonello Migliozzi, Marina Maura Calandrelli

**Affiliations:** 1 Council for Agricultural Research and Economics, Research Centre for Olive, Fruit and Citrus Crops, 81100 Caserta, Italy; rosario.nicoletti@crea.gov.it; 2 Department of Agricultural Sciences, University of Naples ‘Federico II’, 80055 Portici, Italy; migliozz@unina.it; 3 Institute of Biosciences and Bioresources (IBBR), National Research Council (CNR), 80055 Portici, Italy; luigi.demasi@ibbr.cnr.it; 4 Research Institute on Terrestrial Ecosystems (IRET), National Research Council (CNR), 80100 Napoli, Italy

**Keywords:** *Pinus pinea*, *Toumeyella parvicornis*, remote sensing, GIS

## Abstract

For some years, the stone pine (*Pinus pinea* L.) forests of the Domitian coast in Campania, Southern Italy, have been at risk of conservation due to biological adversities. Among these, the pine tortoise scale *Toumeyella parvicornis* (Cockerell) has assumed a primary role since its spread in Campania began. Observation of pine forests using remote sensing techniques was useful for acquiring information on the health state of the vegetation. In this way, it was possible to monitor the functioning of the forest ecosystem and identify the existence of critical states. To study the variation in spectral behavior and identify conditions of plant stress due to the action of pests, the analysis of the multispectral data of the Copernicus Sentinel-2 satellite, acquired over seven years between 2016 and 2022, was conducted on the Domitian pine forest. This method was used to plot the values of individual pixels over time by processing spectral indices using Geographic Information System (GIS) tools. The use of vegetation indices has made it possible to highlight the degradation suffered by the vegetation due to infestation by *T. parvicornis*. The results showed the utility of monitoring the state of the vegetation through high-resolution remote sensing to protect and preserve the pine forest ecosystem peculiar to the Domitian coast.

## 1. Introduction

The stone pine *(Pinus pinea* L.) is a coniferous tree widespread in the Mediterranean basin, particularly along the Italian coast, forming vast evergreen forests [1]. The pine forest of the Domitian coast in northern Campania originated in the last century as a result of reforestation for the management of coastal areas in the provinces of Naples and Caserta [2]. The morphological structure of this coast is typical of the dune environment, which reaches its maximum expression with Mediterranean evergreen scrub formations in the complete ecological succession; in the back of the dune, the herbaceous and shrubby vegetation that primarily covers the ground is replaced by a thick coastal pine forest, consisting mainly of *P. pinea* [3]. This area represents an extremely vulnerable environment, subject to both natural disturbances and strong anthropic pressures [4]. Over the years, pine forests have assumed considerable importance for the performance of ecosystem services and functions of public interest; they have also been perceived as an object of landscape culture and, for all those reasons, are often preserved in protected areas.

This landscape has been progressively altered and transformed due to high anthropic impacts, land-use changes, and the effects of global climate change [5]. Extreme weather events, such as a lack of rainfall followed by prolonged periods of high temperatures, have been particularly damaging and have caused the weakening of entire forest populations; this has favored the proliferation of insect pests in trees with a compromised self-defense capacity [6]. In more recent years, rapid plant decay has been observed in this area [7,8]. Indeed, Garonna et al. (2015) [7] have ascertained that since 2014, the alien pest *Toumeyella parvicornis* (Cockerell), or pine tortoise scale (Hemiptera, Coccidae), native to North America, has been accidentally introduced into Europe, starting in Southern Italy. Today, the pine tortoise scale is a damaging insect pest in several municipalities in Campania (Italy), where it is leading to severe infestations among stone pine trees, *Pinus pinea* L. In 2020, the infestation affected the entire coastal strip up to Salerno, delineating a vast colonized area that contributed to the death of numerous pine trees [9]. This scale insect is multivoltine, with at least three generations per year, favored by the warm Mediterranean climate [10], and overwinters at the adult female stage; therefore, it has a high reproduction rate and potential for spreading. The colonies develop scabs on the twigs and sticky honeydew that is promptly covered by a sooty mold, suppressing photosynthesis and, thus, plant vitality [7]. The timing and duration of these stages may vary as they are influenced by local weather and site conditions [11,12]. The host species *P. pinea* is highly susceptible to this pest, and the wide distribution of regularly planted old-growth pines along thoroughfares has allowed *T. parvicornis* to complete its rapid invasion by moving between adjacent pines or being carried by the wind [7,8,9]. In Italy, the privileged host is the common stone pine [12], while the maritime pine (*Pinus pinaster* Aiton.) and the Aleppo pine (*Pinus halepensis* Mill.), often associated with the stone pine in the same range, seem to suffer negligible damage. Some authors [11,13,14,15,16,17] have reported that pests and pathogens can act in synergy with both mismanagement and adverse climatic factors, anticipating the onset of pinewood decline. Pests can alter the balance of entire forest ecosystems because they often impact them with large population explosions [18] as a consequence of climate change [19]. In this respect, Mediterranean forests are considered among the most vulnerable ecosystems [20]. At the same time, other pests act on now-injured plants, including the pine blastophagus *Tomicus destruens* [21]. As with other bark beetles, *T. destruens* is considered a vector of fungi that can worsen the health conditions of trees, such as *Fusarium circinatum* [13] and *Leptographium wingfieldii* [14,15]. The first step for effective management of forest pests is to ascertain their presence by implementing monitoring programs and developing new methods of observation and impact assessment [22]. In the early stages of pest establishment or at the onset of a disease, although symptoms are not yet visible, plants react through physiological mechanisms, such as reduced photosynthesis, which leads to fluorescence and heat emission variations. Therefore, stressed plants can be identified by spectral signatures different from those of healthy plants [23].

Riley (1989) [24] initially verified that through the visual interpretation of aerial or satellite images, it is possible to identify forest areas affected by plant diseases and pests. Remote sensing can greatly extend the monitoring capacity in time and space by incorporating knowledge of pest–host interactions and showing how damage translates into a signal for detection and mapping [25]. The wide range of remote sensing technologies and the ability to assess disease at an early stage make them an excellent choice for assessing the condition of forests in both space and time at a low cost [26]. Moreover, the use of remote sensing data is motivated by the consideration that remote imagery offers the possibility of collecting open-access data for large geographical areas with medium resolution (10 m) [27]. The most common transformation of remotely sensed spectral data for vegetation classification involves the use of spectral vegetation indices (SVIs). Their use allows for the consistent detection of both gradual and sudden changes caused by pests [28,29]. SVIs are widely used for monitoring, analyzing, and mapping temporal and spatial variations in vegetation structure and certain biophysical parameters [30]. They are an algebraic combination of reflectance values acquired by satellite sensors at different wavelength ranges, calculated pixel by pixel over the entire image [31]. They exploit the typical behavior of vegetation of having a low reflectivity in the visible, with absorption in the red band, and a strong reflectivity in the near-infrared due to the presence of the internal structures of the vegetation [32]. SVIs are simple and effective measurement parameters used to indicate the vegetation cover of the Earth’s surface and the growth status of crops in the remote sensing field [33]. SVIs enable the presence of vegetation on the Earth’s surface and its temporal variation to be captured [34]. Many studies have reported the use of SVIs to map soil dynamics and degradation [35], to differentiate vegetated areas from non-vegetated areas [36], and to monitor vegetation [37] and drought [38]. In other research, they have been used to study vegetation phenology [39], to monitor the health of a crop [40], and to estimate its productivity [41] by highlighting possible problems that reduce photosynthesis (e.g., water stress, disease, fire). Our study was carried out with the visual comparison of satellite images between different years and the help of SVIs that analyze the health status of plants. Through the observation of changes in the spectral structure of vegetation associated with natural biotic factors, i.e., pest infestation, this research aims to highlight how forest monitoring can provide the necessary knowledge to allow targeted interventions to preserve forest ecosystems from irreversible damage.

## 2. Materials and Methods

### 2.1. Description of the Study Area

**Figure 1:**
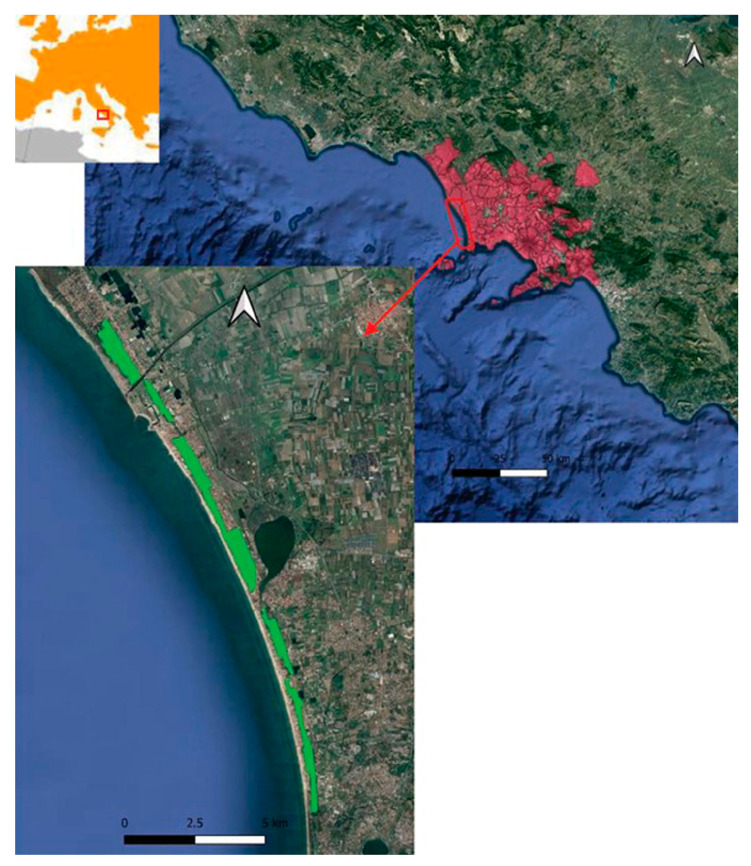
Study area (green-colored) and spread of *Toumeyella parvicornis* (red-colored) in pine forests of Campania.

The Domitian coastline, in northern Campania (Italy), has a length of about 70 km and is characterized by wide, shallow, and sandy beaches [42]. It is characterized by morphologies connected to coastal and river dynamics and, in some cases, to anthropic activity [43]. The latter is due to different actions that have occurred over time [44]. The territory under study involved the southern portion of this stretch of coastline, which is 20 km long and has an area of about 6 km^2^ (Figure 1). This is a formation made up of approximately 90% of stone pine (*Pinus pinea* L.) growing in association with the maritime pine (*Pinus pinaster* Aiton.), the Aleppo pine (*Pinus halepensis* Mill.), the holm oak (*Quercus ilex* L.), and other plants of the Mediterranean scrub [1]. Attacks by the alien pest *T. parvicornis*, recorded in Campania for several years, have caused entire portions of the pine forest to decay [7,8]. Our study area overlaps with the area studied by Garonna et al. [7,8], who identified and determined the specific type of parasite.

In particular, the topsoil is mainly made up of domestic pine, either pure or in association with maritime pine and Aleppo pine, with planting layouts of 2 m × 3 m, 2 m × 4 m, and 3 m × 3 m. In some areas, the stone pine is found in association with the holm oak, which comes to dominate the topsoil in the northern portions of the pine forests belonging to the National Nature Reserve of Castel Volturno. In pine forests, it is common to find polycormic pine plants with 1–3 even-aged trunks, a structure conditioned by excessive density due to the lack of silvicultural interventions (thinning and pruning), the action of sea winds, and, sometimes, the passage of fire [3].

### 2.2. Field Surveys

Field investigations were necessary for the visual interpretation of cover types from satellite images to identify healthy and diseased vegetation, to analyze the spatial and temporal characteristics of tree wilting, and to test for new outbreaks. Although the canopy visible from satellite imagery is clearly identifiable, it is not possible to determine whether tree discoloration is caused by pests or is associated with other phenological causes [45]. To address this issue, teams were sent to the field to check the reasons for the discoloration of these trees. From the visual survey carried out in the pine forest of the study area, clear signs of pest activity were detected on the trees or parts of them [3] (Supplementary Materials). The field surveys allowed us to define the structure of the pine forest, which has very close planting layouts (2 × 3, 2 × 4), with trunks of very different diameters, and stumps with two or three trunks, twisted and inclined in many cases. These structures are evidently conditioned by excessive density and the lack of silvicultural interventions (thinning, subdivision). The presence of holm oak and Aleppo pine was often found within these stone pine communities [3].

### 2.3. Remote Sensing by Sentinel-2 Satellite Images

Multispectral images of the Sentinel-2A satellite were downloaded from Sentinel’s Scientific Data Hub after free registration. The satellite, equipped with a multispectral sensor (Multispectral Instrument—MSI) [46], provides images of the Earth’s surface corrected by light pollution generated by the atmosphere [47,48]. The high spectral resolution of the sensor enables the capture of the effects of weak or inconspicuous diseases and pests at an early stage or in complex scenarios [45]. The Sentinel-2 MSI [47] acquires 13 spectral bands ranging from visible and near-infrared (VNIR) to short-wave infrared (SWIR) wavelengths along a 295 km orbital swath, with a spatial resolution of 10 m, 20 m, and 60 m depending on the band [48,49] (Table 1). Two main products are generated by the satellite mission: Level-1C provides orthorectified top-of-atmosphere (TOA) reflectance, with cloud and land/water masks and sub-pixel multispectral registration included; Level-2A provides orthorectified atmospherically corrected bottom-of-atmosphere (BOA) reflectance, with sub-pixel multispectral registration [47].

In the present study, images acquired monthly on similar dates from 2016 to 2022 were used, choosing images with a cloud cover of less than 10% (Table 2). The images were acquired in May, which represents the most favorable period, for the growth of the green cover of *P. pinea* plants, coinciding with flowering, and for obtaining cloud-free images [50].

### 2.4. Data Processing in GIS

The open-source software QGIS “Semi-automatic Classification Plugin” (SCP) [51] was used to visualize and process the satellite data in GIS. The Sentinel-2 data selected for the study were processed at the L2A level, except for the data for 2016 and 2017, which were processed at the L1C level. Therefore, these latter images were corrected with the ESA Sen2Cor 2.11 processor and brought from the L1C TOA (top-of-atmosphere) level to the L2A BOA (bottom-of-atmosphere) level. From the Corine Land Cover 2016 (CLC2016) Land Use Land Cover (LULC) project [52], the coniferous forest layer was imported into the GIS environment, from which the pine forest extent of the study area was defined. To simplify the analysis and be consistent with the aim of the work, the study was conducted only on the part occupied by the pine forest, thus excluding the other vegetation and land-use types. Therefore, with the geoprocessing operation, the vegetation index rasters were cropped according to the extent of the study area.

### 2.5. Spectral Vegetation Indices 

To analyze the health status of vegetation areas using the spectral wavelength bands acquired by the MSI sensor, four widely used SVIs from recent studies were selected [53,54,55]. The Raster Calculator tool in QGIS version 3.16.9 was applied to the orthoimages of each spectral wavelength band for analysis. For each SVI, the raster’s histogram with the software QGIS was calculated. This allowed us to view the frequency distribution of pixel values in the raster.

#### 2.5.1. NDVI (Normalized Difference Vegetation Index)

NDVI [56] is one of the most widely used vegetation indices; it was calculated according to the following formula:

NDVI = (NIR − Red)/(NIR + Red)

where NIR is the reflectance in the near-infrared and Red is the reflectance in the visible red [57] to represent plant pigmentation and chlorophyll content [58]. They correspond to bands B8 and B4 of Sentinel-2, respectively. Increased reflectivity in the red coupled with decreased reflectivity in the near-infrared indicates that chlorophyll content decreases while damage to the cell structure of stressed plants increases [32]. NDVI values are limited in the range from −1 to 1, but vegetation will have values between 0.1 and 0.8 (>0.2 for vegetation in good condition); negative values correspond to the presence of water masses, while the one close to zero (<0.2) corresponds to bare soil or rock, but also an absence of vegetation due to drought, fire, or pests [59]. NDVI uses the NIR reflectance of vegetation to create a single value that roughly reflects the photosynthetic activity occurring in a pixel; all visible ranges were captured by the satellite camera as bands, through which vegetation characteristics were extracted. This results in a raster image in which each pixel defines an NDVI value. NDVI is a value that does not remain constant throughout the year. Coniferous evergreen forests have a constant trend toward high NDVI values all year, unlike deciduous forests [31]. The interpretation of the absolute value of the NDVI is highly informative, as it allows for the immediate recognition of areas with development problems. It was then necessary to identify the minimum and maximum values of NDVI by carrying out normalization, associating the red color with the minimum value and green with the maximum value. The areas in red are those that are under stressful conditions.

#### 2.5.2. NDMI (Normalized Difference Moisture Index)

NDMI [60] detects moisture levels in vegetation using the combination of near-infrared (NIR) and short-wave infrared (SWIR) spectral bands, according to the following formula:

NDMI = (NIR − SWIR)/(NIR + SWIR)

where NIR and SWIR correspond to bands B8 and B11 of Sentinel-2, respectively. This index is a reliable indicator of water stress in crops. The SWIR spectral channel is sensitive to the water content of the vegetation. On the other hand, the NIR band collects light reflectance from the internal leaf structure and leaf dry matter content. When combined, the accuracy of vegetation water content data becomes much higher. Furthermore, NDMI is a better indicator of deforestation than NDVI due to a less abrupt decrease in values [61]. It can only have values between −1 and 1. Water stress is indicated by negative values approaching −1, while those close to +1 indicate water stagnation.

#### 2.5.3. NDRE (Normalized Difference Red Edge)

The NDRE index [62] is a modified version of the NDVI, which is more sensitive to variations in chlorophyll content. This index is calculated using the following formula:

NDRE = (NIR − RE)/(NIR + RE)

where NIR and RE are the near-infrared and red edge reflectance bands, corresponding to bands B8 and B5 of Sentinel-2, respectively. The scale ranges between −1 and 1. It is important to know how to correctly interpret the values [63].

Values from −1 to 0.2 indicate bare soil.

Values from 0.2 to 0.6 can be interpreted as unhealthy plants.

Values from 0.6 to 1 are good values, indicating healthy plants.

In this way, it was possible to have a reference to highlight the plant communities in good condition and those currently in a state of degradation.

#### 2.5.4. NBR (Normalized Burn Ratio)

The method to calculate this index [64] is similar to the other SVIs, combining the use of near-infrared (NIR) and short-wave infrared (SWIR) wavelengths, according to the following formula:

NBR = (NIR − SWIR)/(NIR + SWIR)

In Sentinel-2, they correspond to bands B8 and B12, respectively. SWIR is a subset of the infrared band of the electromagnetic spectrum, covering wavelengths between 1 and 2.5 μm, and can often provide a better image than visible light images. This index is used to map areas affected by fires. In this study, it was used in analogy to map desiccation caused by pest action and define severity, understood as the degree to which an area has been altered or disrupted due to a lack of chlorophyll activity. Healthy vegetation has a very high near-infrared reflectance and a low reflectance in the short-wave infrared, in contrast to burnt/dried areas that have a low near-infrared reflectance and a high reflectance in the short-wave infrared band. Typically, NBR is obtained from the pre-fire and post-fire images; the pre-fire image is subtracted from the post-fire image to create the differentiated NBR image, dNBR. Thus, the new image obtained is used for damage severity assessment. Areas with higher dNBR values indicate more severe damage, while areas with negative dNBR values show higher vegetation productivity. The damage suffered by the vegetation was classified according to the severity ranges proposed by the United States Geological Survey (USGS) [65]. 

## 3. Results and Discussion

**Figure 2:**
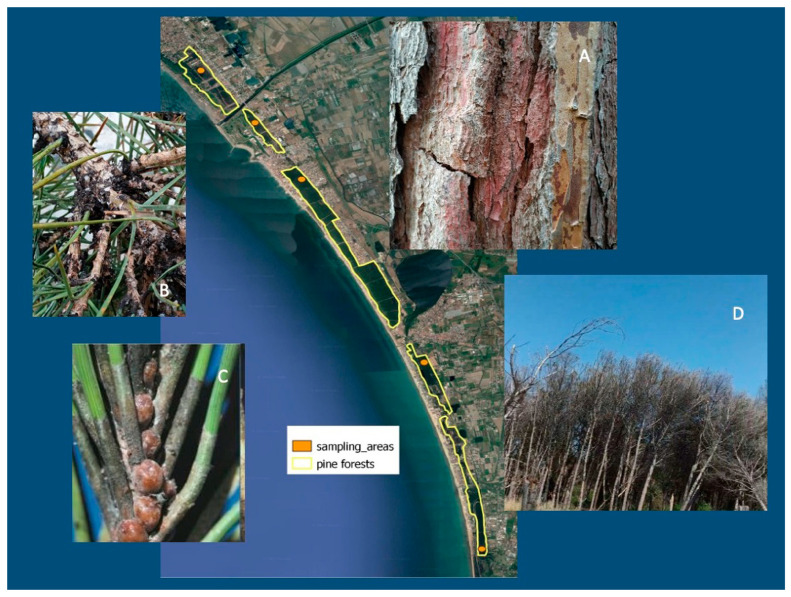
Sampling areas, signs of pest infestation, and tree condition in pine forests. (**A**) Traces of redness on the trunk of the pine tree; (**B**) formation of sooty crust on branches; (**C**) *Toumeyella parvicornis* (Cockerell). Adult female specimens on a pine twig [8]; (**D**) current status of the pine forest.

For some years, pine forests in the study area have been in danger of conservation due to insect aggression [7]. Field teams sent out to validate the reason for the discoloration of these trees determined that the primary attack by *T. parvicornis* caused serious damage to the terminal shoots of the branches [3]. The infested twigs and shoots may fall to the ground below when still green due to strong winds or heavy rain, or they may dry out directly on the crown [66]. Within a few years, the pine tree weakens and becomes more susceptible to attacks by other pests [67]. The finding of numerous terminal shoots at the foot of the pine trees is a symptom of a serious risk for pine populations in that locality [68]. From the visual survey carried out in the pine forest of the study area, signs of pest activity were detected (Figure 2; Supplementary Materials).

**Figure 3:**
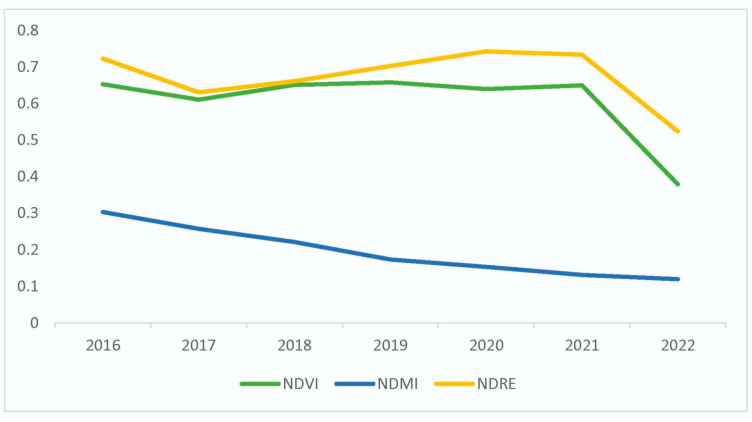
Trends of the NDVI, NDMI, and NDRE indices in the 2016–2022 period.

Previous studies [25,26,28,29] have shown that the values of the SVI decrease when there are factors that may affect the health of the vegetation because they are influenced by the amount of chlorophyll contained in the plants. The trend of the three SVIs used in Figure 3, i.e., NDVI, NDMI, and NDRE, was determined by considering data acquired on similar dates in May using the pixels from the study area of 6 km^2^ (see Section 2). It was observed that their value decreases with time in the considered period (Figure 3); on the field, this trend is visually highlighted by the gradual dieback of the plants (Figure 2D). More in detail, the average values of the NDMI progressively decreased, while the other two indices, NDVI and NDRE, decreased over the long period, showing relative trends that were very similar to each other. The variations observed in the indices are related to the gradual drying due to the reduced photosynthetic activity of the pines infected by *T. parvicornis.*

Based on Figure 3, the first damage began to be clearly visible in 2017 by direct comparison with the 2016 data, even if the infestation had already been present with less evident signs since 2014 [3,7]. The real decline in the field throughout the considered areas is particularly evident from 2022, with a manifest and irreversible drying highlighted by the low values of NDVI and NDRE in Figure 3, as also reported by Somma et al. (2023) [12]. In this last year, the vegetation indices suffered a sudden fall, as also confirmed by the recent analysis of D’Amico et al. (2023) carried out on the entire extent of the Campania region [4]. However, it must be underlined that the small fluctuations in specific trends observed in Figure 3 can be due to the use of average values of indices for the entire territory and to the spectral characteristics of the different wavelengths; in fact, in smaller sectors, the decline has already been present to a lesser extent since 2017.

**Figure 4:**
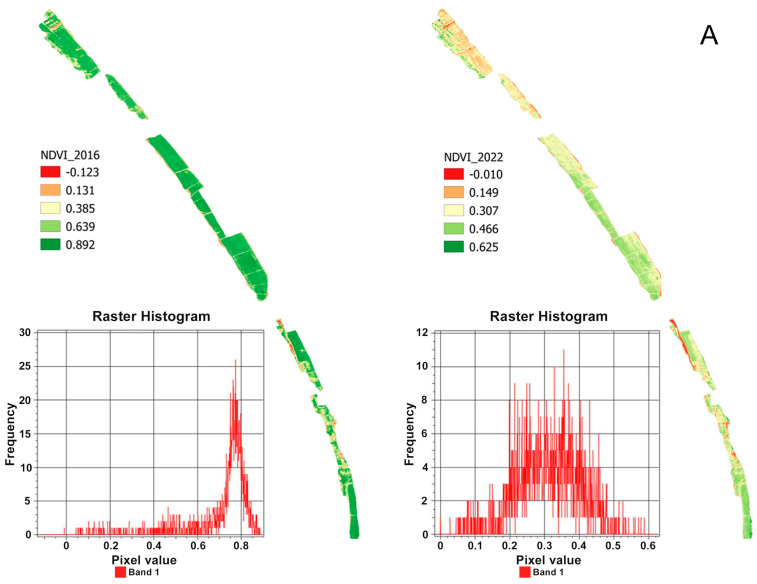

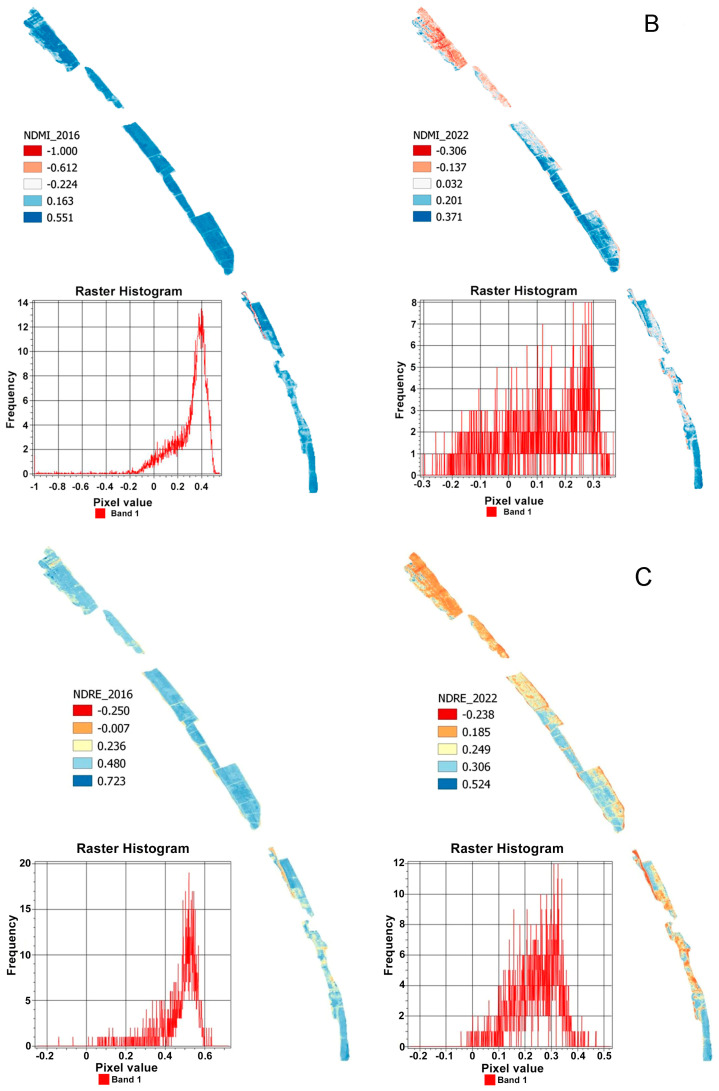
The figure shows SVI raster images for the years 2016 and 2022 with the relative histograms. (**A**) NDVI. (**B**) NDMI. (**C**) NDRE.

SVI analyses referring to the satellite images from 2016 to 2022 show a substantial change in the photosynthetic capacity. In particular, the associated histograms show a decrease in the maximum values and, at the same time, a shift in pixel frequency toward lower values, which indicates a decreased presence of chlorophyll (Figure 4). These results are shown by the trend of the NDVI, as indicated by the histograms associated with the images in Figure 4A, in which, from 2016 to 2022, there was a notable contraction in the maximum value of the index from 0.892 to 0.625. More in detail, in 2016, the histogram showed a greater frequency of values above 0.7, while in 2022, the values were mainly restricted to the 0.2–0.4 range. The same trend was recorded by the NDMI and NDRE (Figure 4B,C), in which the histograms highlighted a variation in the frequency of pixels associated with the index values.

The mean values of each index were highest on 20 May 2016 (NDVI: 0.705, NDMI: 0.303, NDRE: 0.474) and lowest on 10 May 2022 (NDVI: 0.346, NDMI: 0.119, NDRE: 0.239). This indicates that the pines have been diseased and have gradually withered. As for the trends of each vegetation index, the average values changed by about 0.4 for NDVI (0.359), 0.2 (0.184) for NDMI, and more than 0.2 (0.235) for NDRE over the period considered. While the average values of the NDMI index decreased steadily, the other two indices, although decreasing over the long term, had different trends; this seems to be due to the characteristics of the spectral wavelength bands, which were used to calculate the vegetation indices [69]. The decrease in the indices is due to the reduced photosynthetic activity of the pines, as the foliage, which gradually desiccates, is no longer able to absorb light energy [70]. The results of the analysis of the vegetation indices used in the study were represented with raster images. The relative histograms were associated with each SVI (Figure 4).

Data analysis showed that the decrease in the NDVI value for the study period indicates significant changes in the quality of the vegetation cover of the pine forest, which tends to worsen. Similarly, the decrease in the NDMI index indicated that the tree canopy has reduced its water content. The NDRE index also decreased, especially in the northern part of the study area, where the attack began, and, consequently, has been affected by the pest for a longer time. The field survey, reinforced by the existing bibliography on the study area [3,7,8,12], made it possible to demonstrate that this phenomenon is exclusively linked to the pest infestation. The field activity was carried out in sample areas representative of the degradation status of the coastal pine forest dominated by *P. pinea*. A summary example of the checks is shown in the Supplementary Materials. The results of the SVI analysis combined with the field survey gave a first indication of the vegetation’s response at short- and long-time intervals to the *T. parvicornis* attack.

**Figure 5:**
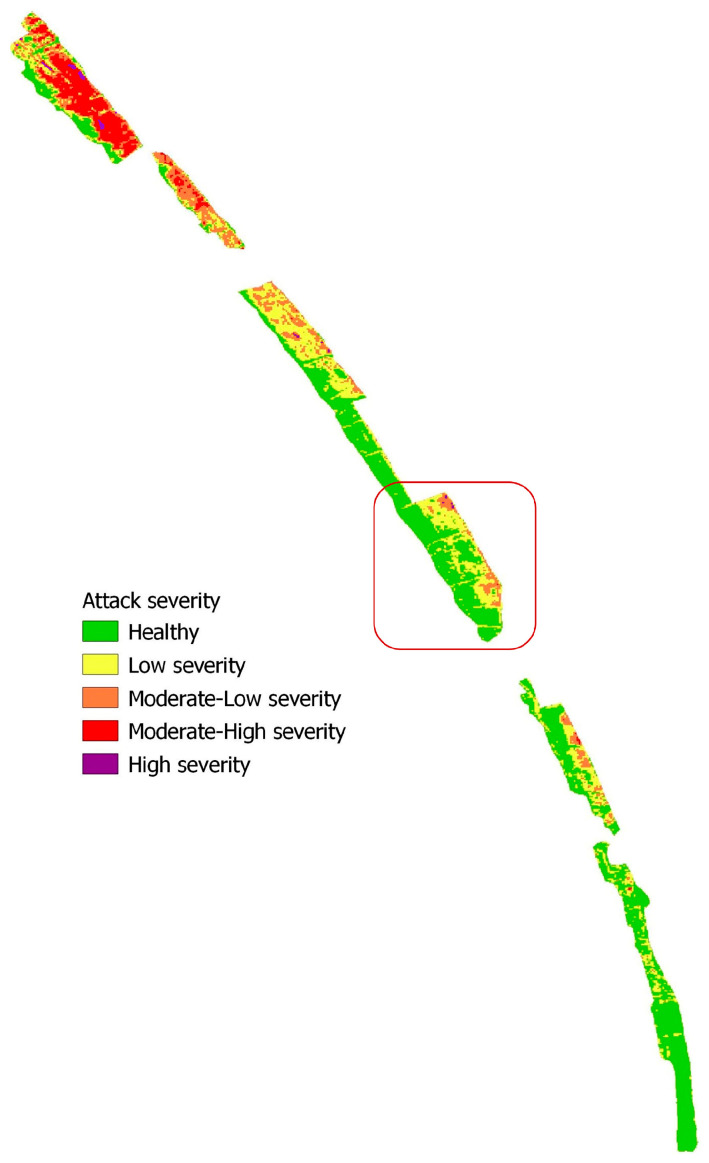
Map of the damage severity caused by the attack of parasites based on dNBR. The figure highlights the sectors most damaged by parasitic activity. In the box, the sector of the study area is shown in the panchromatic image of Figure 6.

In addition, the NBR index was determined to visualize on a map the severity of the damage suffered by the pine forest over time (Figure 5).

**Figure 6:**
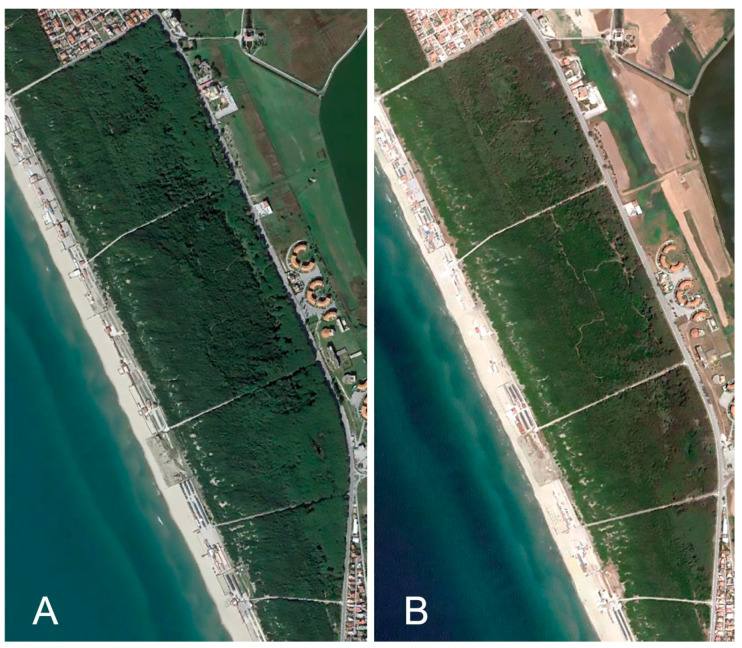
Satellite images of a sector of the study area. The images taken in 2016 (**A**) and 2022 (**B**) are compared; they allow us to make a quick visual comparison on the state of decline of the pine forest.

Here, in agreement with the results of the other SVIs, the differentiated NBR image (dNBR) consistently showed a higher degree of damage, corresponding to the minimum values of NDVI, NDMI, and NDRE, and reduced damage, where the highest values of the other SVIs are recorded. This study showed that SVIs are suitable for providing general information on the phenological state of the canopy when a phenomenon that reduces plant photosynthetic activity, such as parasitic activity in this case, is in progress. This observation, validated after field investigations, provided evidence of the presence of pests, such as reddening of the bark, terminal shoots at the foot of trees, and the presence of sooty crust on needles and branches. Furthermore, in terms of temporal characteristics, the study showed that it is possible to also use a single image per year with comparable dates in the considered period to differentiate healthy areas from wilted ones (Figure 6A,B).

**Figure 7:**
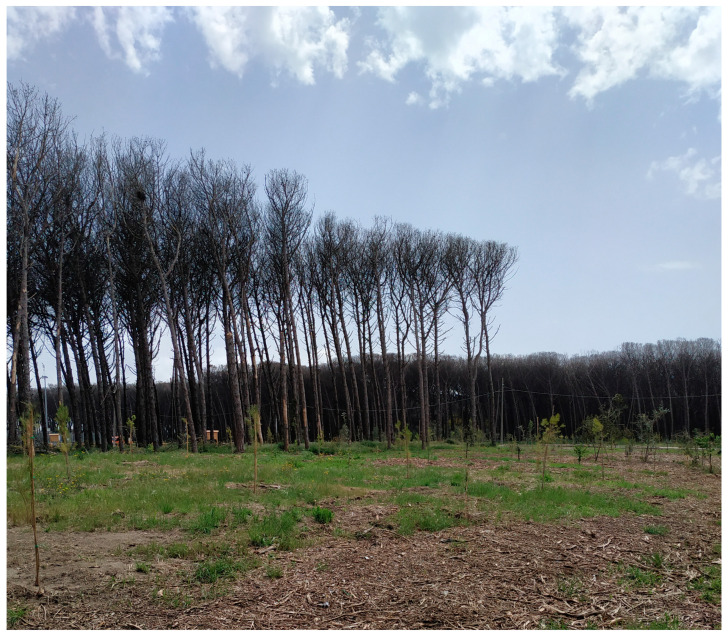
Current status of the pine forest and first reforestation interventions with *P. pinea* and *Q. ilex*.

The observation of panchromatic images from 2016 to 2022 showed that the first signs of pest attack, with consequent aerial desiccation, were visible in 2017. While forest pest damage can be detected in a single remote sensing image, damage monitoring requires images from two or more dates [25]. In recent years, due to climate-related environmental changes, large areas of *P. pinea* forest have been continuously exposed to pest damage that has reduced vigor to the point of causing entire forest areas to disappear [71]. Since the first outbreak in 2014, *T. parvicornis* has rapidly spread on the Domitian coast of Campania and has heavily attacked *P. pinea* trees widespread in these forests. Several factors may have contributed to the development of pest populations at such an impressive scale [72]. The increasingly frequent desiccation of pines due to *T. parvicornis* attacks has already brought considerable changes to the coastal ecosystem and radically altered the typical landscape of Mediterranean coastal belts. For this reason, various interventions from public institutions have been planned, and a spatial–temporal control strategy against *T. parvicornis* is becoming mandatory. Lack of timely eradication intervention and spread monitoring has caused part of the pine forest to dry out. Environmental regeneration works are underway with the aim of restoring a healthy, resilient forest that can be used by citizens and tourists, which will also have repercussions on the economic and social profile [3]. The interventions consist of cutting dead plants and replanting new specimens using the same plant species that characterize the landscape naturalness in a typical Mediterranean coastal environment (Figure 7). From a long-term perspective, more natural and less monospecific forests should be planted to reduce vulnerability to parasites, increase biodiversity, and counteract parasite attacks with biological control [73].

Monitoring of at-risk areas has become indispensable to follow the extent of the pest damage spread [74] and to preserve territories that are still healthy by implementing appropriate practices to prevent pest reproduction and spread [9], e.g., immediate removal and destruction of plants or parts of host plants infested by the parasite, control of the presence of the specified parasite and treatments to prevent its further spread, and limitations on movement outside the infested area for material coming from felling. These containment actions aim to create an environment that enhances the development and self-defense capabilities of attacked plants and, at the same time, discourages the establishment of pests. In the event of a pest attack, visual control from the ground can be integrated with careful monitoring of the aerial part, especially if the infested forest is difficult to access. Remote monitoring has proven to be efficient and is effectively supportive of field activities. The results of the present study have shown that using SVIs from satellite images can support monitoring health status in forest environments, as also shown by other recent research [45,75,76].

## 4. Conclusions

Global change and increased human activities induce changes in the habitats of various organisms, including plants and animals, resulting in reduced ecosystem stability and increased insect infestations and pathogen epidemics. To minimize impacts and control them effectively, adequate systems for monitoring forest health are essential, in which remote sensing tools play an increasingly important role, as well as supporting field analyses. The presented approach confirms the reliability and effectiveness of remote sensing in providing useful and timely information that should be better exploited to monitor the health status of the pine forests. Sentinel-2 data have proven to be a crucial source of information. According to our results, a growth trend in disturbed areas has been going on over the years, with a sharp increase in 2022, as also highlighted in other recent studies [4], from which a very critical situation for Mediterranean pinewood dominated by stone pine emerged.

Starting from the numerous observations carried out around the world [77], the results of global climate models and current knowledge on ecological interactions suggest that there will be an increase in parasite infestations in the future; this may soon lead to a reduction in the geographical distribution of *P. pinea*, locally and globally [70]. These issues represent a challenge for forestry research in the coming years. The study recommends continuous monitoring using high-resolution satellite imagery to identify critical events and take timely actions to ensure forest conservation.

## Figures and Tables

**Table 1:** Spatial and spectral characteristics of Sentinel-2 satellite.

Spectral Band	Central Wavelength (nm)	Spatial Resolution-GSD (m)
B1: Ultra Blue (coastal, aerosol)	443	60
B2: Blue	490	10
B3: Green	560	10
B4: Red	665	10
B5: Red-edge 1	705	20
B6: Red-edge 2	740	20
B7: Red-edge 3	783	20
B8: Near-infrared (NIR)	842	10
B8A: Near-infrared narrow	865	20
B9: Water vapor	945	60
B10: Short-wave infrared (SWIR)	1375	60
B11: Short-wave infrared (SWIR)	1610	20
B12: Short-wave infrared (SWIR)	2190	20

**Table 2:** List of Sentinel-2 images used in this study.

N	Identifier Data
1	S2A_MSIL1C_20160523T095032_N0202_R079_T33TVF_20160523T095404
2	S2A_MSIL1C_20170518T095031_N0205_R079_T33TVF_20170518T095716
3	S2A_MSIL2A_20180526T100031_N0208_R122_T33TVF_20180526T161700
4	S2B_MSIL2A_20190523T095039_N0212_R079_T33TVF_20190523T124054
5	S2A_MSIL2A_20200522T095041_N0214_R079_T33TVF_20200522T122957
6	S2A_MSIL2A_20210507T095031_N0300_R079_T33TVF_20210507T151839
7	S2A_MSIL2A_20220515T100031_N0400_R122_T33TVF_20220515T141508

## Data Availability

Data are contained within the article and Supplementary Materials.

## Supplementary Materials

The following supporting information can be downloaded at https://www.mdpi.com/article/10.3390/plants13020182/s1.
