# Supplementary material for: Analysis of Dieback in a Coastal Pinewood in Campania, Southern Italy, through High-Resolution Remote Sensing
Source: Plants (Basel). 2024 Jan 9;13(2):182. doi: 10.3390/plants13020182 (PMC10818449; doi:10.3390/plants13020182)

SAMPLE PLOT 5

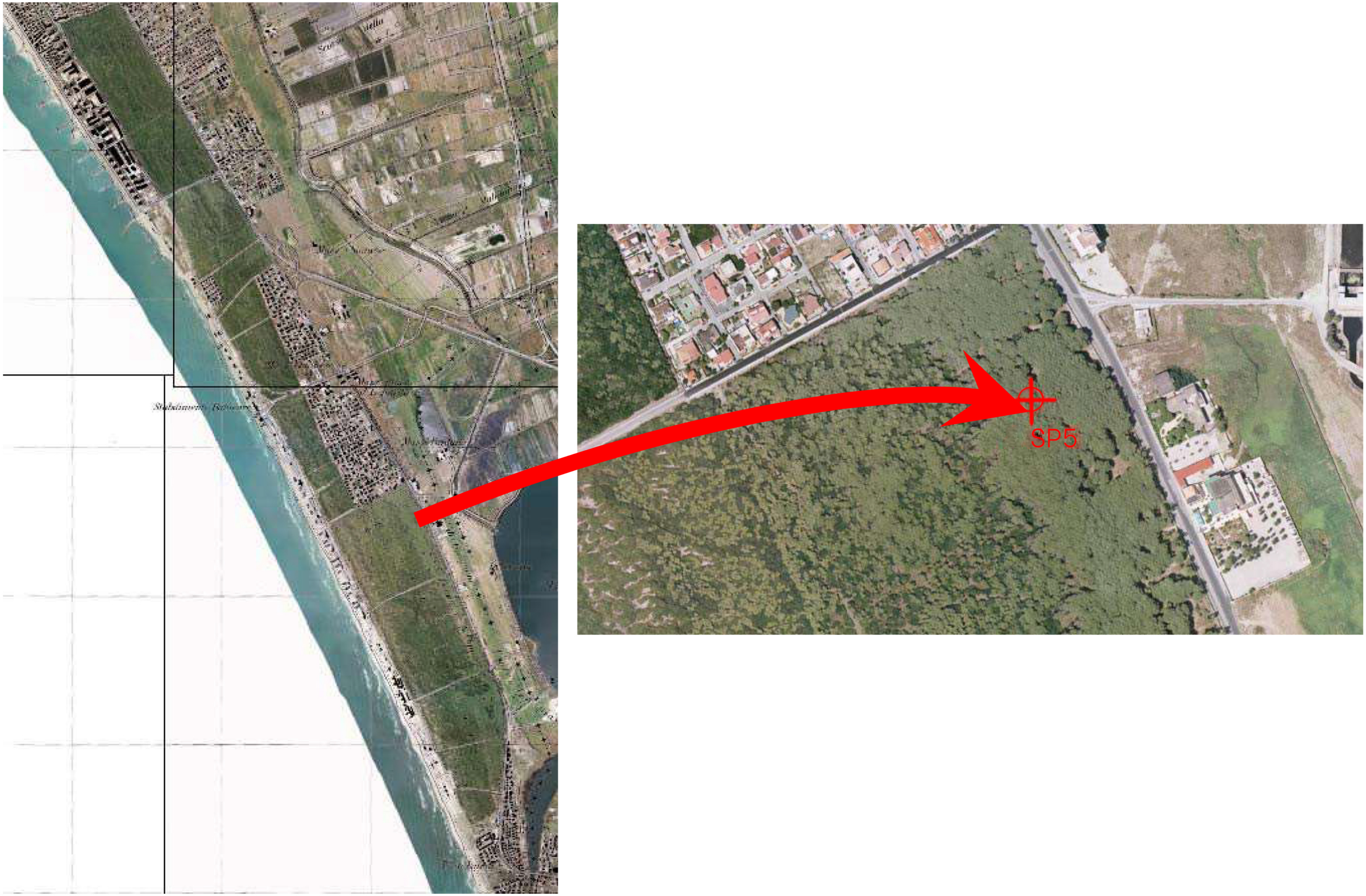

TOPSOIL ANALYSIS

TOPSOIL DESCRIPTION

Even-aged forest Pinus pinea L. dominated. In the part of the thinned high forest and adequate distances between the plants, the largest diameters are found (> 40cm), while in the non-thinned area the diameters are on average 24 cm. Green foliage present in the upper 1/4 of the stem, semi-transparent, many stems are inclined, especially in the area not thinned towards the Domitiana state road. Polycormic stone pine plants present. Structure conditioned by excessive density caused by the lack of silvicultural interventions (thinning and spreading) as well as by the action of sea winds. Dense layer of undecomposed litter on which a sparse herbaceous layer develops consisting mostly of Smilax aspera, holm oak seedlings, Rubia peregrina, Asparagus acutifolius. Renewal of Pinus pinea absent.

TOPSOIL TRANSECT REPRESENTATION

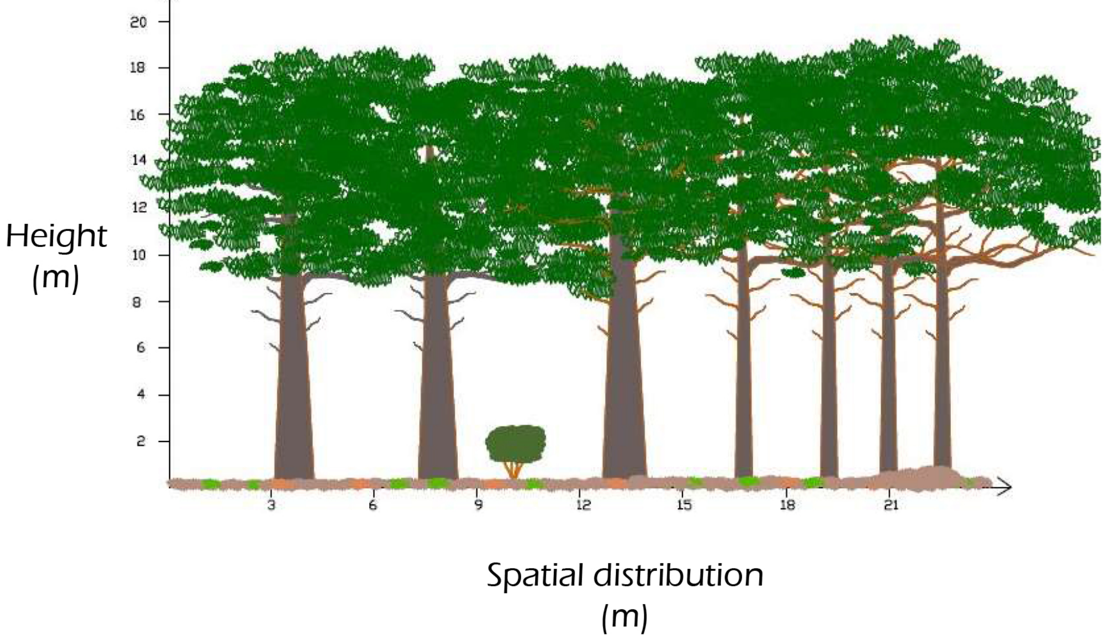

TOPSOIL PHOTOGRAPHIC REPRESENTATION

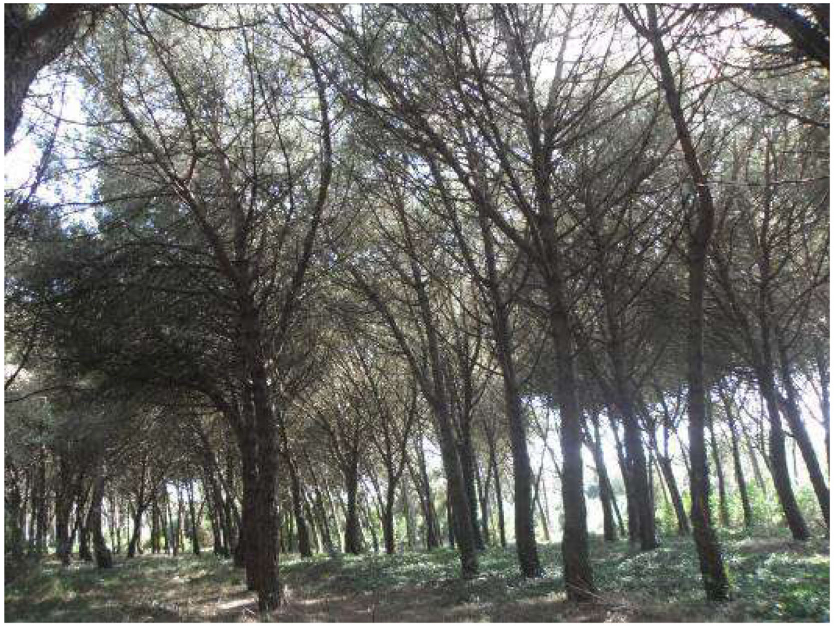

DENDROMETRICS PROCESSING

SP5								
Pine forest Sector 5	Location	Sup.(ha)	Aspect	Alt. (m)	Slope Avg	Max	Age	
Diameter at 1,30 m (cm)	Species, Plants number							
	Pinus pinea						tot	tot/ha
5							0	0
10							0	0
15	3						3	24
20	24						24	191
25	25						25	199
30	13						13	103
35	4						4	32
40	4						4	32
45	3						3	24
50	4						4	32
55	3						3	24
60							0	0
65								
70								
75								
80								
85								
90								
95								
100								
Plants	83	0	0	0	0	0	83	
tot/ha	660	0	0	0	0	0		660
Dead Plants	11	0	0	0	0	0	11	
Dead p./ha	88	0	0	0	0	0		88
Stumps	0	0	0	0	0	0	0	
stumps/ha	0	0	0	0	0	0		0
Dead stumps	0	0	0	0	0	0	0	
Dead st./ha	0	0	0	0	0	0		0
Basal Area	5,82	0,00	0,00	0,00	0,00	0,00	5,82	
Basal Area/ha	46,3	0,0	0,0	0,0	0,0	0,000		46,28

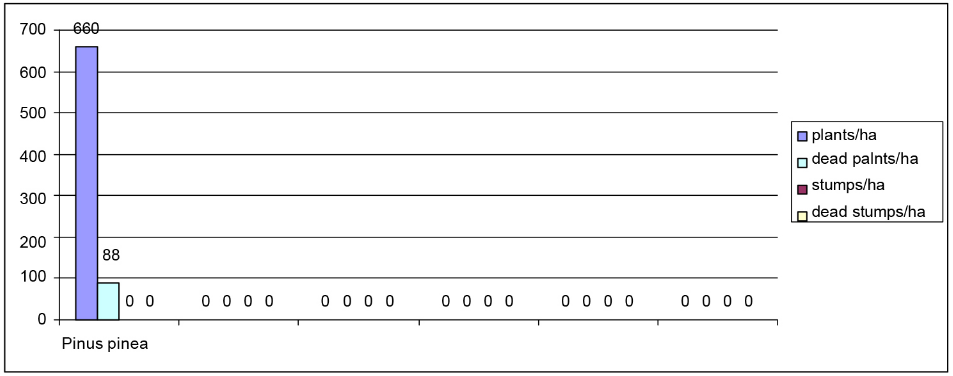

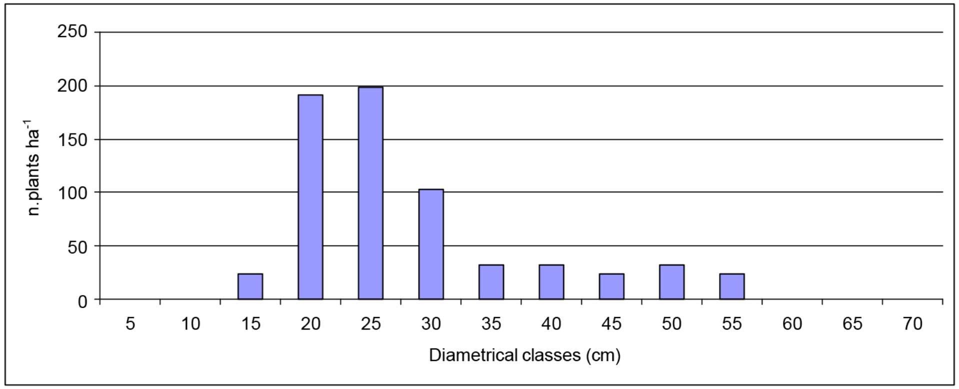

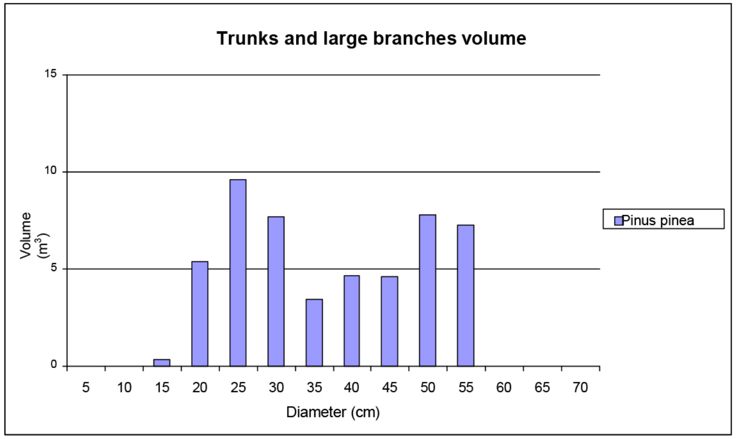

Supplement: Supplementary file 1 [file plants-13-00182-s001.zip › plants-2688835-supplementary.pdf]
